# Metagenomic and phylogenetic analyses reveal gene-level selection constrained by bacterial phylogeny, surrounding oxalate metabolism in the gut microbiota

**DOI:** 10.1128/msphere.00913-24

**Published:** 2025-05-13

**Authors:** Sromona D. Mukherjee, Mangesh Suryavanshi, John Knight, Dirk Lange, Aaron W. Miller

**Affiliations:** 1 Department of Cardiovascular and Metabolic Sciences, Cleveland Clinic, Cleveland, Ohio, USA; 2 Department of Urology, University of Alabama at Birmingham, Birmingham, Alabama; 3 The Stone Centre at VGH, Department of Urologic Sciences, University of British Columbia, Vancouver, British Columbia, Canada; 4 Department of Urology, Glickman Urological and Kidney Institute, Cleveland Clinic, Cleveland, Ohio, USA; University of California at Davis, Davis, California, USA

**Keywords:** oxalate, gut microbiome, dietary toxins, complex systems, selfish gene, gene radiation

## Abstract

The gut microbiota is critical for neutralizing dietary toxins. Oxalate is a toxin commonly produced by plants to deter herbivory and is widely consumed in the human diet. Excess levels of systemic or urinary oxalate increase risk of multiple urologic and cardiometabolic diseases. The current study employed multiple amplicon-based and shotgun metagenomic methodologies, alongside comparative phylogenetic analyses, to interrogate evolutionary radiation surrounding microbial oxalate degradation within the human gut microbiome. In conservative genome-based estimates, over 30% of gut microbial species harbored at least one oxalate-handling gene, with the specific pathways used dependent on bacterial phylum. Co-occurrence analyses revealed interactions between specialist genes that can metabolize oxalate or its by-products, but not multi-functional genes that can act in more than one oxalate-related pathway. Specialization was rare at the genome level. Amplicon-based metagenomic sequencing of the oxalate-degrading gene, formyl-CoA transferase (*frc*), coupled with molecular clock phylogenetic analyses are indicative of rapid evolutionary divergence, constrained by phylum. This was corroborated by paired analyses of non-synonymous to synonymous substitutions (dN/dS ratios), which pointed toward neutral to positive selection. Sequence similarity network analyses of *frc* sequences suggest extensive horizontal gene transferring has occurred with the *frc* gene, which may have facilitated rapid divergence. The *frc* gene was primarily allocated to the Pseudomonodota phylum, particularly the *Bradyrhizobium* genus, which is a species capable of utilizing oxalate as a sole carbon and energy source. Collectively evidence provides strong support that, for oxalate metabolism, evolutionary selection occurs at the gene level, through horizontal gene transfer, rather than at the species level.

**IMPORTANCE:**

A critical function of the gut microbiota is to neutralize dietary toxins, such as oxalate, which is highly prevalent in plant-based foods and is not degraded by host enzymes. However, little is known about the co-evolutionary patterns of plant toxins and the mammalian gut microbiota, which are expected to exhibit features of an evolutionary arms race. In the current work, we present molecular evidence that microbial genes for oxalate degradation are highly prevalent in humans, potentially driven by extensive horizontal gene transfer events. Phylogenetic analyses reveal that oxalate-degrading genes are under a positive selection pressure and have historically undergone rapid diversification events, which has led to diverse ecological strategies for handling oxalate by gut bacteria. Collectively, data shed light on potential evolutionary relationships between the diet and the gut microbiota that occur relatively independently of the mammalian host.

## INTRODUCTION

The microbiota and host, collectively termed a holobiont (1), can be considered a complex system complete with extensive metabolic redundancy, cooperation, and hierarchy in distinct host-microbe, microbe-host, and microbe-microbe interactions (2). The gut microbiota is thought to have evolved primarily through microbe-microbe competition within the host environment, while the host has evolved to keep the microbiota from overwhelming host defenses (2). In such an environment, resources for gut bacteria are derived primarily from the diet (3). As such, there is a strong selective pressure, defined as environmental factors that influence the survival and reproductive success of individuals within a population (4), to utilize as much of the nutritional resources of the diet as possible, setting the stage for fundamental ecological and evolutionary processes to occur over short- and long-term time scales (5). As such, the gut microbiota provides a historical record of environmental exposures over ecological to evolutionary timescales and can provide fundamental insights into how the environment shapes survival strategies of its inhabitants. One important question in ecology and evolution is “What is the unit of selection?” Traditionally, the species/taxon has been thought to be the unit of selection. However, in the last several decades, there has been more of a focus on genes as the unit of selection (reviewed in reference 6). Another important question is focused on how specialist vs generalist ecological strategies evolve (7, 8). Again, specialization/generalization could occur at either the gene or species level.

To probe these questions within the host-microbe holobiont, an ideal study system would involve the utilization of resources that are rare, difficult to access, or are toxic to other bacteria. Such a system would place a bottleneck on the evolution of phenotypes that can effectively utilize the resource, allowing such evolutionary features to be more apparent than those universally distributed among all or most gut bacteria. Dietary toxins, defined as secondary metabolites produced by plants to deter herbivory (9), that inhibit the proliferation of bacteria fit these criteria. Among toxins, the simple dicarboxylic acid, oxalate, stands out as a particularly effective molecule to study the ecological and evolutionary processes of the gut microbiota. Oxalate is produced by many plants as a means to deter herbivory and is, thus, found in a variety of plant-based foods (10, 11). Oxalate is known to inhibit the growth of many gut bacteria (12, 13). However, while mammalian hosts do not have the enzymatic machinery to degrade oxalate (14), there are species of gut bacteria, such as *Oxalobacter formigenes,* that can utilize oxalate as a carbon and energy source, through relatively simple metabolic pathways (15). As such, oxalate exposure provides a selective pressure on the gut microbiota to remove a toxin for bacteria, provides a carbon and energy source for growth within a unique and difficult to access niche, and removes any host contribution that would remove this toxin, which would otherwise alleviate some of the selective pressures on the bacteria. This toxin is also detrimental to the host when absorbed into circulation, leading to multiple chronic conditions, such as kidney stones, chronic kidney disease, or cardiovascular disorders (16). As such, this dietary toxin also puts pressure on the microbe-host evolutionary relationship, which is thought to be of little relevance compared to microbe-microbe or host-microbe relationships (2).

**Fig 1:**
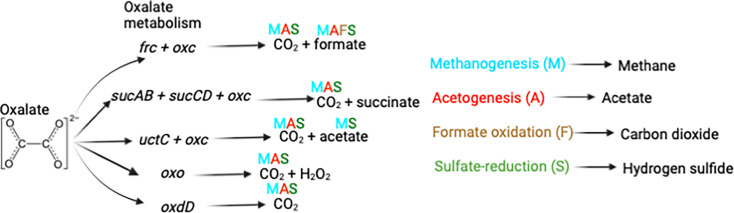
The bacterial oxalate-degrading pathways assessed in the current study. The by-products of each pathway are labeled with the downstream metabolic pathways that they could be a substrate for (color-coded). Right—downstream metabolic pathways that can utilize oxalate metabolism by-products and the by-products of those pathways.

In gut bacteria, there are multiple metabolic pathways to oxalate degradation. One gene, *oxIt,* encodes an oxalate-formate transport protein (17). There exists a pair of genes, formyl-CoA transferase (*frc*) and oxalyl-CoA decarboxylase (*oxc*), that first converts oxalate to formate and an oxalyl-CoA and then converts the oxalyl-CoA to CO_2_ and a formyl-CoA (18, 19). In this reaction, the formyl-CoA can be substituted by a succinyl-CoA, encoded by the genes *sucAB* and *sucCD*, which produces a succinate instead of a formate molecule (20). The formyl-CoA can also be substituted with an acetyl-CoA, encoded by the *uctC* gene, to produce acetate instead of formate (21). Oxalate oxidase, encoded by the *oxo* gene, converts oxalate and oxygen to H_2_O_2_ and 2 CO_2_ (15). Finally, the oxalate oxidoreductase pathway, encoded by the *oxdD* gene, converts oxalate and oxidized ferredoxin to 2 CO_2_ and reduced ferredoxin (22) (Fig. 1). Importantly, formate can act as either a toxin or a resource for bacteria (23–27), dependent on taxonomy, similar to oxalate. In fact, several by-products of oxalate metabolism can be used in diverse microbial metabolic pathways (Fig. 1) and, thus, put ecological and evolutionary pressure on the gut microbiota.

The objectives of the current study were to probe the evolutionary ramifications of dietary oxalate exposure in the gut microbiota of humans and laboratory mice. The current study was designed to delineate hypotheses that historical oxalate exposure drove evolution at either the gene or taxon level. Here, we assume that gene-level selection is indicated by (i) the same genes, performing the same function, present across taxonomic boundaries, indicative of horizontal gene transfer events (i.e., the presence of the *frc* gene in multiple taxa); (ii) co-occurrence of genes within a community; (iii) adaptation of the same genes for multiple, but similar metabolic pathways (i.e. formate dehydrogenase in acetogenic, methanogenic, and formate oxidation metabolic pathways), indicative of generalism at the gene level; and (iv) the fluid radiation of individual genes across taxonomic boundaries. In contrast, taxon-level selection would be defined by (i) different genes present between diverse taxa that perform the same function (i.e. the evolution of *sucAB* to perform the same function as *frc,* demarcated by taxonomic boundaries); (ii) the co-occurrence of species within a community; (iii) the integration of multiple genes to perform similar functions in similar metabolic pathways; and (iv) the rigid radiation of individual genes along taxonomic boundaries.

Multiple metagenomic approaches were used to investigate our hypotheses, which included (i) comparative shotgun metagenomic analysis and genome reconstructions in a human cohort of 70 participants with or without urinary stone disease (USD) in a clinical case control study and (ii) amplicon-based metagenomic sequencing of the *frc* and 16S rRNA genes in 27 human participants and 16 laboratory mice, coupled with molecular clock phylogenetic analyses, non-synonymous: synonymous ratios (dn:ds) of *frc* gene variants, and sequence similarity network (SSN) analyses of *de novo* metagenomic data and UniProt reference sequences. Amplicon-based data were derived from a clinical intervention study with oxalate-degrading probiotics (prior to the intervention) or a controlled diet animal study to determine the impact of oxalate on mice, respectively. Collectively, the data used here, drawn from multiple independent human and murine sources, provide rich and robust data sets to gain fundamental insight into evolutionary processes surrounding oxalate metabolism. The results of this study shed light on fundamental evolutionary processes that include the emergence of metabolic redundancy, co-opting metabolic genes as a means of specialization or generalization in a highly competitive environment, horizontal gene transfer, and the relevance of dietary toxins for microbial evolution in the gut microbiota.

## MATERIALS AND METHODS

### Comparative genomic analyses

To delineate hypotheses surrounding gene vs taxon selection surrounding oxalate metabolism, we examined shotgun metagenomic data and full-length, metagenome-assembled, genomes obtained from a previously published shotgun metagenomic data set derived from a cohort of 35 patients with USD and 35 healthy, live-in partners (accession no. SRR15021121–SRR15021153 and SAMN21355140–SAMN21355217). Genome assembly for these data has been described in detail previously (28). Briefly, shotgun metagenomic data were assembled in metaSPAdes (29), binned to genomes in Autometa (30), and oriented and scaffolded in CAMSA (31) using the closest taxonomic relatives downloaded from NCBI. Genome gaps were filled using raw data for each sample in Abyss-Sealer (32). The completeness of each genome was by quantifying the number of universal single copy genes in a database of 41 known single copy genes (33). Purity was calculated through the total number of universal single copy genes compared to the number of unique single copy genes. Genomes were considered full length if they were greater than 80% complete and 90% pure, following current standards (34). Reconstructed genomes underwent gene extraction and annotation using PROKKA. Subsequently, genes were screened for known oxalate-degrading genes or genes associated with CO_2_ and formate metabolism, which are two common by-products of oxalate metabolism. Previous research suggests that formate, specifically derived from oxalate metabolism, can be utilized in additional pathways (24, 35). Specifically, genes in the acetogenic, methanogenic, sulfate-reducing, or formate oxidation (AMSF) pathways were screened. The selection of genes for each pathway was based on their annotation in KEGG metabolic pathways (36). Some genes, such as formate dehydrogenase, were present in multiple metabolic pathways. Genomes were classified by the oxalate or AMSF genes present, along with their taxonomy. Subsequently, high-quality shotgun metagenomic data derived from USD patients and healthy controls were mapped separately to the annotated genomes and genes, using the BWA aligner (37), to generate taxonomic and oxalate/AMSF (OAMSF) pathway profiles in the phyloseq (38) package in R statistical software (39). Differential abundance analyses of OAMSF pathways between healthy and USD individuals were conducted in the DESeq2 R package (40). Following the default protocol, a false discovery rate correction was used (FDR < 0.05) for significance (40). Additionally, correlations between OAMSF gene counts along with counts from genomes mapped either to taxonomy or to the presence of OAMSF pathways from the shotgun metagenomic data were conducted with the Sparcc algorithm in the SpiecEasi R package (41) and networks were visualized in Cytoscape (42), annotated by gene function or by the presence of genes in the OAMFS pathways. Only correlations with FDR *P*-values < 0.05 and correlation values either <−0.4 or >0.4 were included for subsequent analysis. Additionally, the number of significant correlations between unique gene variants or genomes was normalized to the total number of gene variants or genomes being compared, to normalize for differences in the number of genes within a category and plotted on a heatmap. Co-occurrence networks were generated between all pairwise comparisons of individual gene variants or genomes and were not matched to themselves.

### Phylogenetic analysis of the *frc* gene

Analysis of amplicon-based metagenomic paired (16S rRNA and *frc* gene) sequencing of human-derived and laboratory mouse-derived stool DNA was based on previously published data (accession no. SAMN38839607–SAMN38839718 and SAMN38777353–SAMN38777654). Amplicon sequencing of the 16S rRNA gene followed standard protocols using primers targeting the V4 hypervariable region of the 16S rRNA gene (515F and 806R primers) (43). For the *frc* gene, primers were developed based on the frc-171F (5′-CTSTAYTTCACSATGCTSAAC-3′) and frc-306R (5′-GDSAAGCCCATVCGRTC-3′), which has previously been validated to target the *frc* gene from a broad diversity of oxalate-degrading bacteria (44). Human-derived data came from 27 healthy participants (151 samples total) as part of a clinical intervention study to investigate the impact of *Oxalobacter formigenes* colonization on urinary oxalate levels, with samples taken prior to *O. formigenes* administration. Mouse-derived data came from a study of mice with a global knockout of the APOE gene to determine the impact of oxalate and antibiotics on oxalate homeostasis. Samples were collected longitudinally in APOE^−/−^ mice (*n* = 16) before any intervention, one week after basal or oxalate diets were started, and after eight weeks on their respective interventions (48 samples total). The 16S rRNA and *frc* gene sequences were independently assigned to amplicon sequence variants (ASVs) in dada2 (R software) and imported into phyloseq (R software) for analysis (38, 45). To ensure the quality of *frc* gene annotation, BWA aligner was used to map the *frc* gene ASVs to the UniRef90 gene database for unbiased annotation (37, 46). The UniRef90 database contains all the reference genes from the UniProt database, clustered at 90% sequence homology. A phylogenetic analysis, using a neighbor-joining algorithm (phangorn package in R software), was used to validate the *frc* and non-*frc* annotations, as previously described (47). This unbiased mapping step identifies any mis-amplified sequences that are not likely to be actual *frc* genes. Non-*frc* annotations were removed from subsequent analysis.

Taxonomic annotation of the *frc* genes was carried out by mapping sequences to a database containing over 45,000 full-length prokaryotic genomes from the NCBI with BWA aligner, ensuring an unbiased method for taxonomic assignment. A selection of *frc* sequences was cross-referenced with the NCBI database to ensure correct taxonomic assignment. However, the 16S rRNA gene, for which there are well-established tools for taxonomic annotation, was annotated using the Silva v138.1 SSURef and NCBI 16S rRNA databases in dada2 (R software), as done previously (48, 49).

### Molecular clock analysis of the *frc* gene

To calculate evolutionary radiation in *frc* gene sequences, a phylogenetic tree *frc* ASVs derived from high-throughput sequencing was constructed using the Reltime algorithm in Mega11 software (50, 51). This algorithm estimates time trees from molecular sequences in lineages with variable evolutionary rates. RelTime provides a relative timeclock, based on relative divergence events, when specific data to calibrate absolute time in evolutionary divergence is absent. This method has been shown to be accurate in simulated and real-world, calibrated data (50). The RelTime phylogenetic analysis allowed for the determination of relative evolutionary divergence among the sequences to deduce evolutionary relationships among the taxonomically-annotated *frc* gene ASVs. The RelTime analysis was conducted with all unique *frc* gene ASVs and after clustering at 97% sequence homology. Clustering was done with the CD-hit algorithm (52).

Phylogenetic analysis was repeated with the paired 16S data, clustered at a 97% sequence homology, enabling a direct comparison between *frc-*containing species and species in the complete microbiome. This comparative analysis between the 16S and *frc* genes was performed to provide an independent assessment of redundancy in oxalate metabolism.

### dN/dS selective pressure analysis

Calculation of the number of non-synonymous to synonymous substitutions between pairs of homologous sequences provides an estimate on whether genes are undergoing positive, negative, or neutral selection within a community (53). We calculated the dN/dS ratio for pairwise comparisons of *frc* gene ASVs using MEGA-11 software, using the codon-based *Z*-test for selection (54) with an FDR correction for multiple hypothesis testing, to assess whether the gene is undergoing positive selection (dN/dS > 1), negative selection (dN/dS < 1), or is stable (dN/dS = 1).

### Sequence similarity network analysis

Sequence similarity network analyses provide an alternative method to phylogenetic analysis of aligned sequences and produce a visual representation of sequence clusters or families, which can be applied to different levels of sequence homologies (55). To generate SSN’s for the *frc* gene, protein sequences from amplicon metagenomic data and all protein sequences annotated as *frc* gene from the UniProt database were each, separately mapped to themselves to produce all pairwise metagenomic:metagenomic or UniProt:UniProt *frc* sequence homologies. UniProt genes were used both in their full sequence form and trimmed to match the amplicon region of the metagenomic data, for direct comparison to the metagenomic data. Networks of each data set were generated by trimming all homologous pairs below the thresholds of 50%, 60%, 70%, 80%, and 90% sequence homologies. The resulting networks were visualized in Cytoscape and annotated by taxonomy (42).

## RESULTS

### Proportion of 152 full-length genomes from a single human population that harbor oxalate or AMSF genes

**Fig 2:**
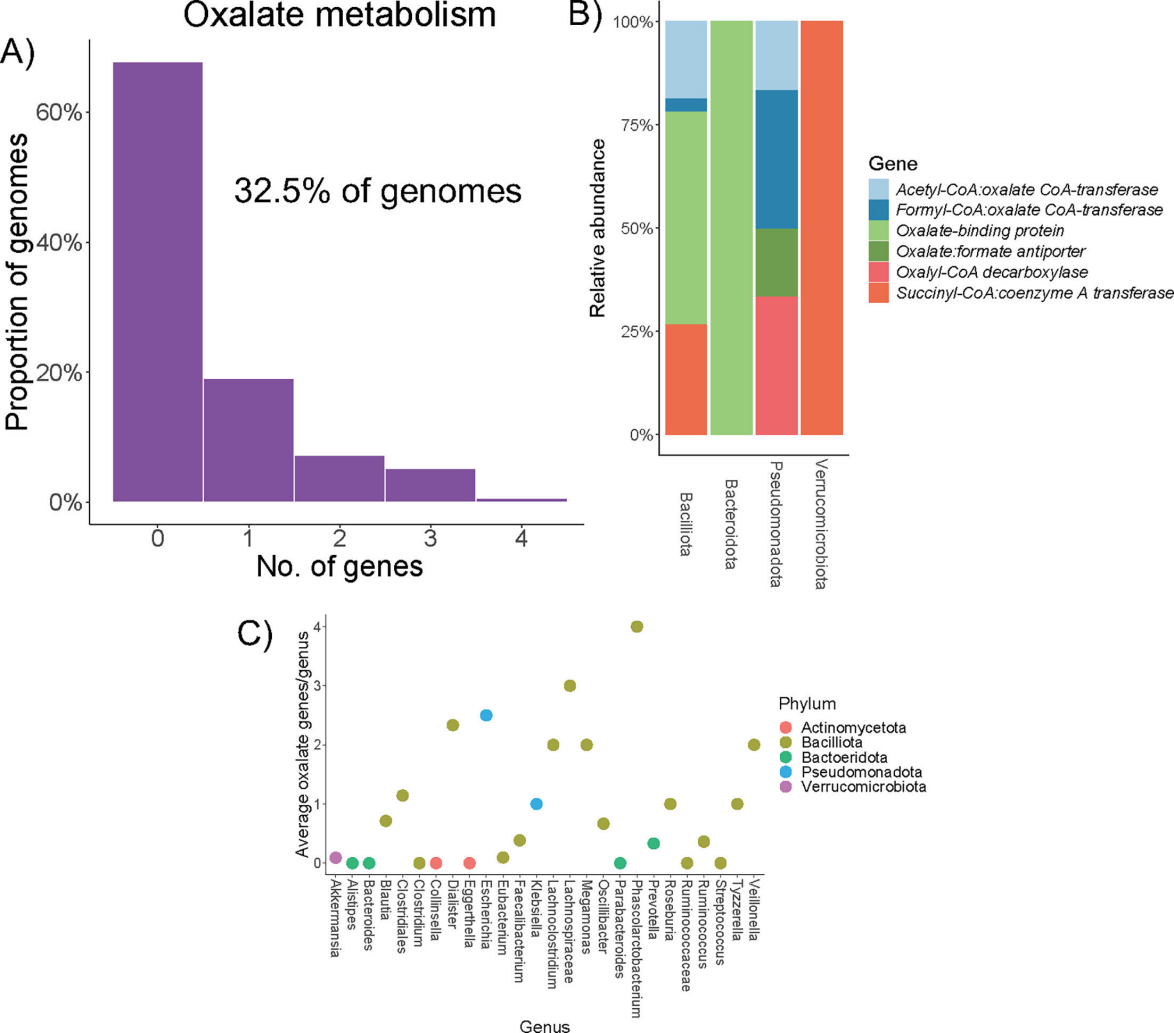
Oxalate-degrading gene distribution in a human population. (A) Distribution of oxalate-related gene number from 152 genomes based on metagenomic data from human stool samples. (**B**) Oxalate-handling gene distribution in the 152 bacterial genomes, by phylum. (**C**) Average number of oxalate-handling genes in each genome by genus.

Based on the presence of oxalate-degrading genes in metagenomic assembled genomes derived from shotgun metagenomic sequencing data of human stool, we estimate that 32.5% of genomes contain at least one oxalate-handling gene (Fig. 2A). Oxalate-binding proteins, which bind calcium oxalate crystals to promote aggregation and influence oxalate degradation (56), were the most represented among genomes being present in 44% of genomes that contained at least one oxalate-handling gene. Of genes directly involved in oxalate degradation, succinyl-CoA, and acetyl-CoA transferases were most abundant, followed by formyl-CoA transferases, oxalyl-CoA decarboxylases, and oxalate-formate antiporters. The distribution of specific oxalate-handling genes largely fell along phylum-level demarcations with the *oxit-frc-oxc* pathway almost entirely contained to the Pseudomonadota phylum, the Verrucomicrobiota phylum only containing *succinyl-CoA* genes, and with the oxalate-binding proteins being relegated to the Baciliota and Bacteroidota phyla (Fig. 2B). Overall, the distribution of oxalate-handling genes was primarily relegated to the Bacilliota phylum, followed by Bacteroidota and Pseudomonadota (Fig. 2C).

In contrast to oxalate degradation, analysis indicates that between 95% and 98% of genomes harbored at least one of each of the AMSF genes, distributed primarily among the Bacilliota and Bacteroidota phyla. The most prevalent genes in these pathways included formate-tetrahydrofolate ligase, phosphoglycerate mutase, a bifunctional oligoribonuclease, and hydroxypyruvate reductase (Fig. S1 to S4). With the exception of methanogenesis, genes for each function largely fell along phylum-level demarcations, similar to oxalate metabolism.

**Fig 3:**
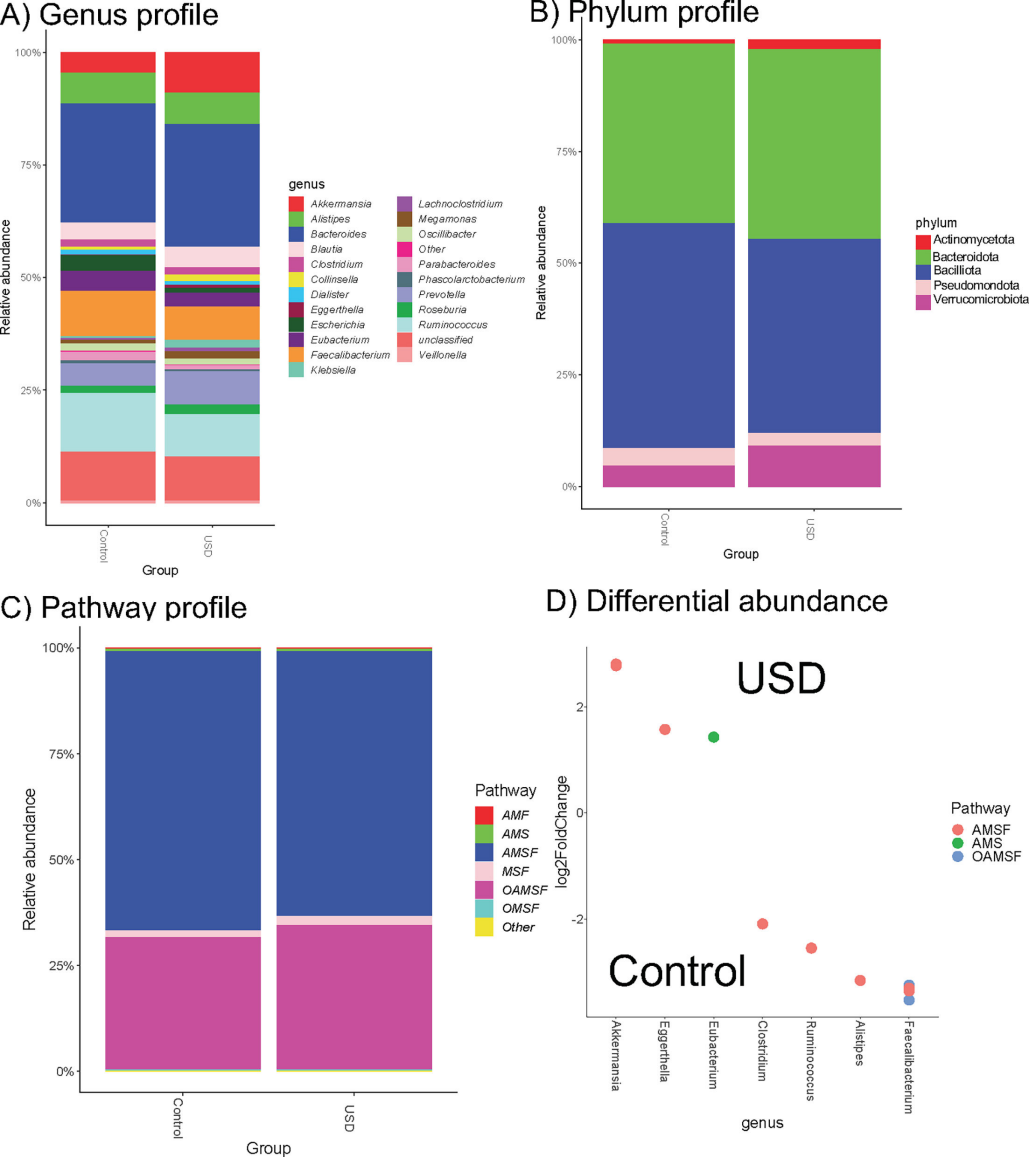
Genomic pathway distribution in individuals with and without USD. (A, B) Genus (**A**) and phylum (**B**) taxonomic profiles between USD and control groups, based on extracted genomes. (**C**) Distribution of OAMSF genes among extracted genomes, in USD and control groups. Pathway combinations that comprised <1% of all genomes are aggregated as “Other.” (**D**) Significant differences in genera between USD and controls, based on a DESeq2 analysis of shotgun metagenomic reads mapped to extracted genomes. Data are annotated with genus and OAMSF pathways. Letters in the legends of panels C and, D indicate metabolic pathways, as follows: O, oxalate-handling; A, acetogenic; M, methanogenic; S, sulfate reduction; F, formate metabolism.

In comparing individuals with or without USD, we found that the two different populations were separated by genomes containing OAMSF pathways. Specifically, individuals without a history of USD had significantly greater levels of reads mapping to genomes with OAMSF pathways compared to USD patients. In particular, individuals in the control group had much greater levels of bacteria from the *Faecalibacterium* genus, which contained oxalate-degrading genes (Fig. 3A through D). Interestingly, in *Faecalibacterium* genome reconstructions, we found that of the 13 *Faecalibacterium* genomes constructed, five harbored oxalate-degrading genes (38.5%). However, of the *Faecalibacterium* strains enriched in controls, 67% harbored oxalate-degrading genes connected to the acetyl-CoA:oxalate CoA transferase pathway, and were all annotated as *F. prausnitzi*.

**Fig 4:**
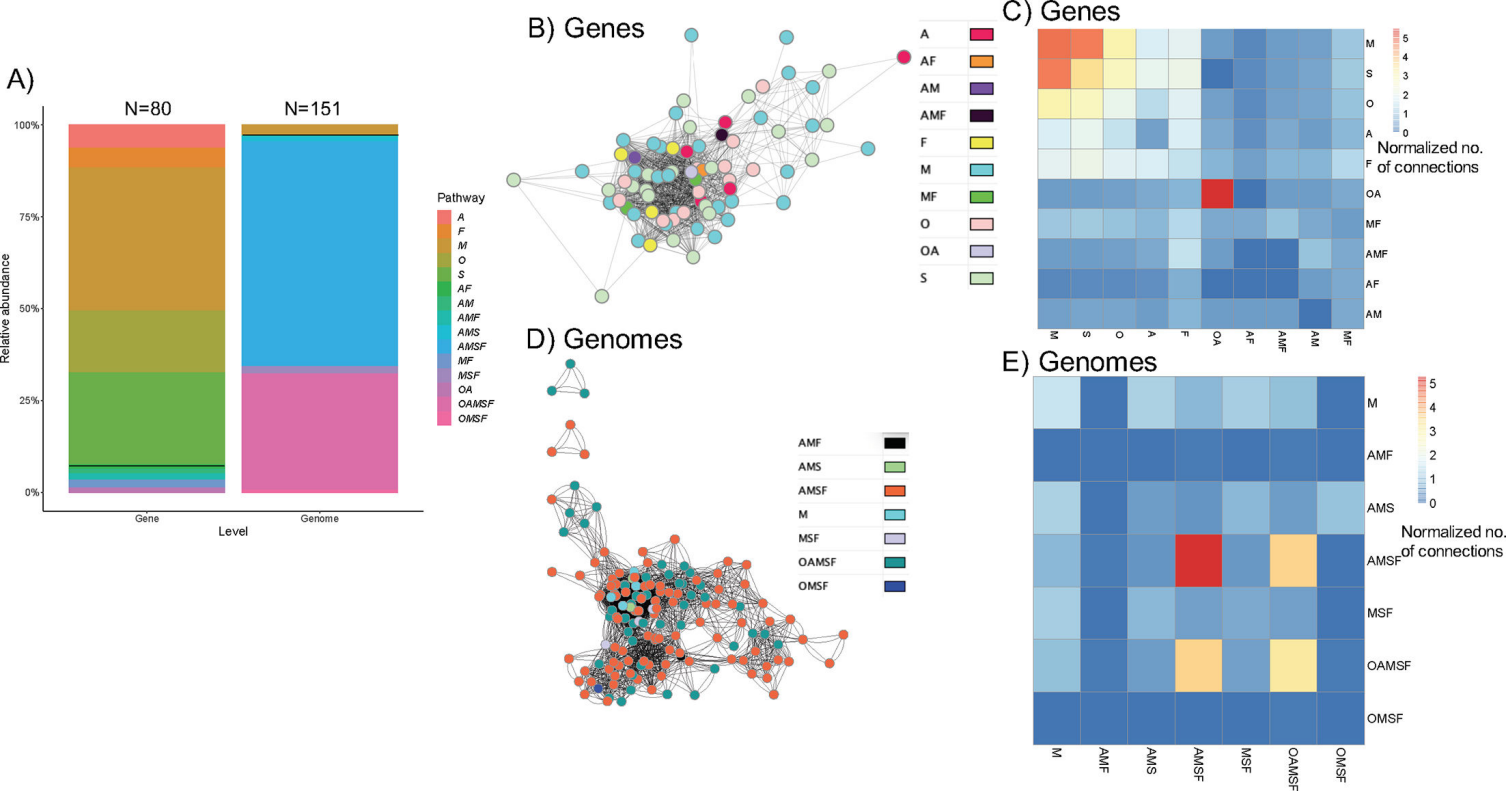
Co-occurrence network analysis of OAMSF genes in metagenomic data. (A) Relative abundance of genes vs genomes annotated to OAMSF pathways. The black line demarcates single function (above line) vs multi-function (below line) genes or genomes relative to OAMSF pathways; (**B**) co-occurrence network among OAMSF genes in shotgun metagenomic data. Network is based on all pairwise Pearson correlations and false discovery rate corrected *P*-values. Network in visualized in Cytoscape. Lines indicate significant positive correlations. (**C**) Heatmap depicting the number of significant, positive correlations between genes annotated to metabolic pathway, normalized to the total number of genes in each OAMSF grouping. (D) Co-occurrence network among OAMSF-containing genomes in shotgun metagenomic data. (E) Heatmap depicting the number of significant, positive correlations between genomes annotated based on the presence of genes in the OAMSF pathways, normalized to the total number of genes in each OAMSF grouping. Letters in the legends indicate metabolic pathways, as follows: O, oxalate-handling; A, acetogenic; M, methanogenic; S, sulfate reduction; F, formate metabolism.

We found that of the 80 unique OAMSF genes identified from the genomes, the majority (92%) were single function genes (Fig. 4A). This is in contrast to the 2.6% of genomes that contained genes pertaining to only one of the OAMSF pathways. Between OAMSF genes in the shotgun metagenomic data, a tight co-occurrence network formed among all genes (Fig. 4B). No significant negative correlations were produced from the data. Quantification of the number of significant positive correlations, normalized to the total number of unique genes being compared, between genes revealed that most significant positive correlations occurred between genes present in only one of the OAMSF pathways and correlations between genes present in multiple OAMSF pathways were sparse (Fig. 4C). Comparing the number of normalized correlations between unique genomes revealed little co-occurrence overall, except between genomes that harbor genes from all or nearly all of the OAMSF pathways (Fig. 4D and E).

### Molecular clock phylogeny of the *frc* gene

**Fig 5:**
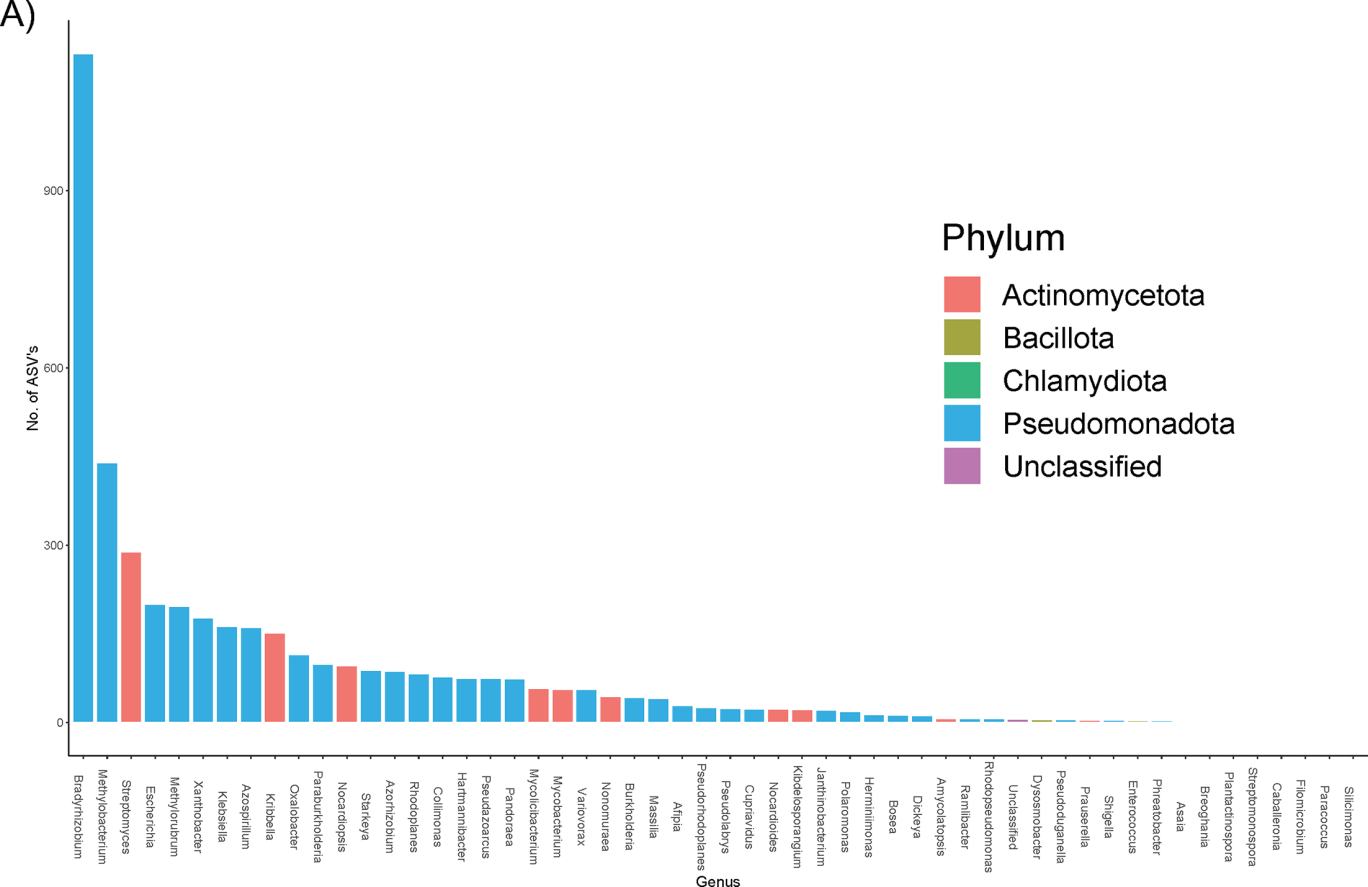
Molecular clock phylogeny of frc gene. (A) Histogram depicting the distribution of the *frc* gene from high-throughput amplicon sequencing, annotated to genus and phylum.

To gain further insight into the relationship between gene- vs taxon-level selection processes, we focused additional analyses on a single oxalate-handling gene (*frc*), using a data set generated through high-throughput sequencing of the gene in human and murine populations. Similar to genome-based results that indicated that the *frc* gene was predominately relegated to the Pseudomonadota phylum, high-throughput sequencing of the frc gene revealed that ASVs also mapped almost entirely to the Pseudomonadota phylum, which includes genera, such as *Bradyrhizobium* and *Oxalobacter*, that can use oxalate as a sole carbon and energy source. In fact, >25% of all unique ASVs mapped to *Bradyrhizobium* genomes. When ranking taxa by number of ASVs per genome, *Oxalobacter* ranked 10th (Fig. 5).

**Fig 6:**
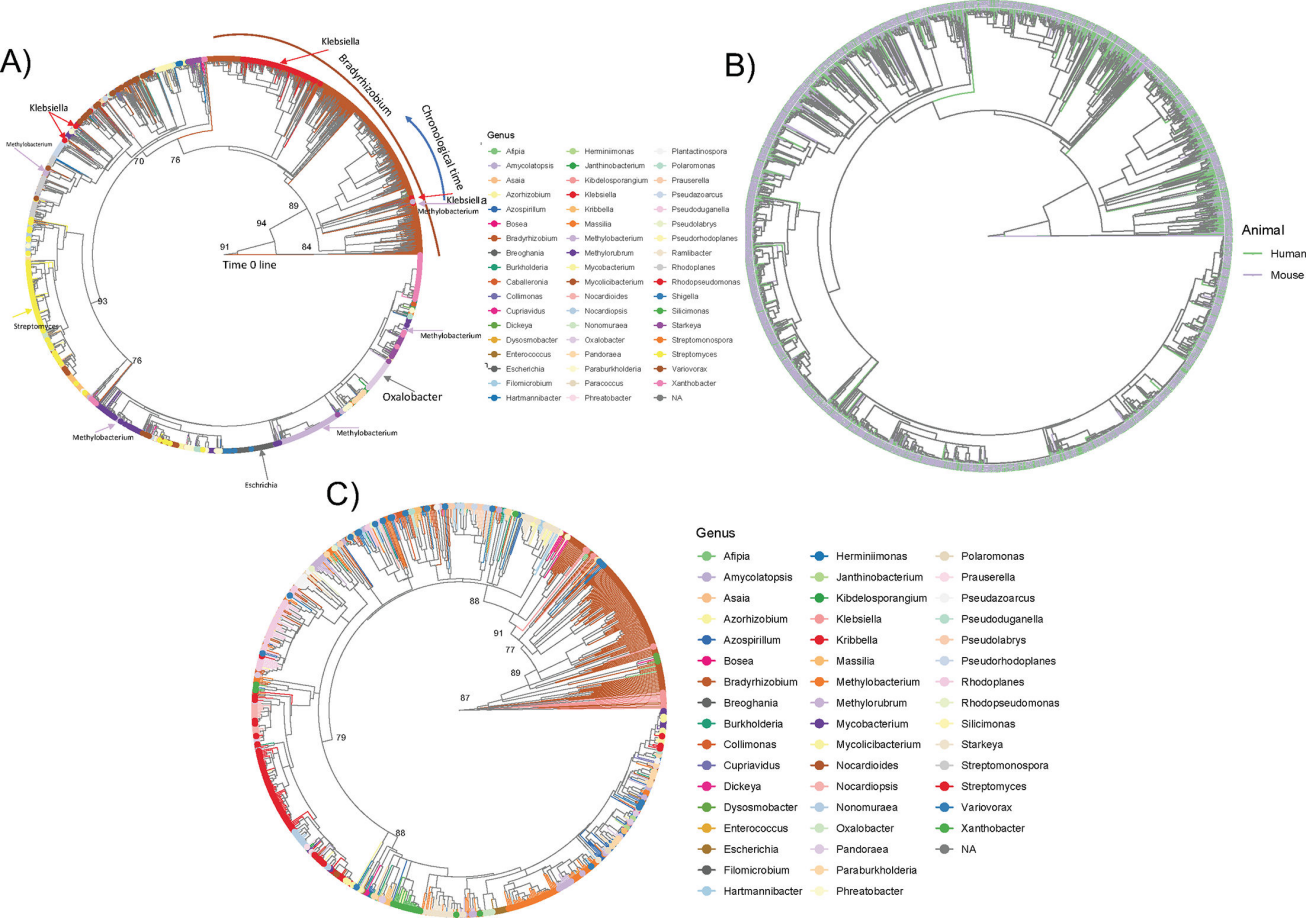
Phylogenetic and diversity analysis of the frc gene in gut microbiota. (A) RelTime phylogenetic analysis based on high-throughput *frc* amplicon sequencing. Direction of chronological time is labeled, and key taxa are highlighted with text and arrows matching the color of the taxa from the legend. Numbers reflect bootstrap values of major branches. (**B**) RelTime phylogenies of *frc* ASVs colored by the host species origins. Bootstrap values are same as in panel **A**. (C) RelTime phylogeny of *frc* ASVs clustered at 97% sequence similarity. Numbers reflect bootstrap values of major branches.

Phylogenetic analysis using the RelTime algorithm, which provides an estimate of relative radiation events of a gene, indicates that the *Bradyrhizobium* genus harbors the more ancestral version of the *frc* gene, while *Oxalobacter* is much more derivative (Fig. 6A). It is apparent that some genera, such as *Bradyrhizobioum*, *Oxalobacter*, or *Streptomyces* acquired the *frc* gene once, which diversified within the genus over time. Other genera, such as *Klebsiella* or *Methylobacterium,* appear to have acquired the *frc* gene on multiple occasions (Fig. 6A). When comparing phylogenies of human vs murine derived *frc* ASVs, there are no apparent demarcations, suggesting frequent migration events between these host populations (Fig. 6B). When the *frc* ASVs were clustered at 97% similarity, the number of unique ASVs decreased by 80%. However, this did not change the overall structure or relative taxonomic relationships compared to the tree with all ASVs, indicative of a robust set of evolutionary relationships (Fig. 6C). When comparing the number of 97% homologous clusters of the *frc* gene to the number of 97% homologous clusters of the 16S rRNA gene, which seeks to normalize varying divergence rates of the two genes, we found that the number of unique species harboring the *frc* gene was 5.7% of the taxonomic units with the 16S rRNA gene. This analysis provides a second estimate for the proportion of the gut microbiota that contains oxalate-degrading genes and is compared to 3.9% of the genomes that contained an *frc* gene (Fig. 2). If we extrapolate these results for all oxalate-degrading genes, we estimate that as much as 42.7% of the human gut microbiota may contain at least one oxalate-handling gene. This estimate is based on comparing the 5.7% figure comparing the number of homologous *frc* vs 16S rRNA clusters and the 3.9% of genomes containing *frc* relative to the 32.5% of genomes containing at least one oxalate-handling gene and provides a range of 32.5%–42.7% of species in the gut that contains at least one oxalate-handling gene. The taxonomic profiles of the gut microbiota in humans are provided in Fig. 4 and for mice in Fig. S5.

### dN/dS ratios of the *frc* gene

**Fig 7:**
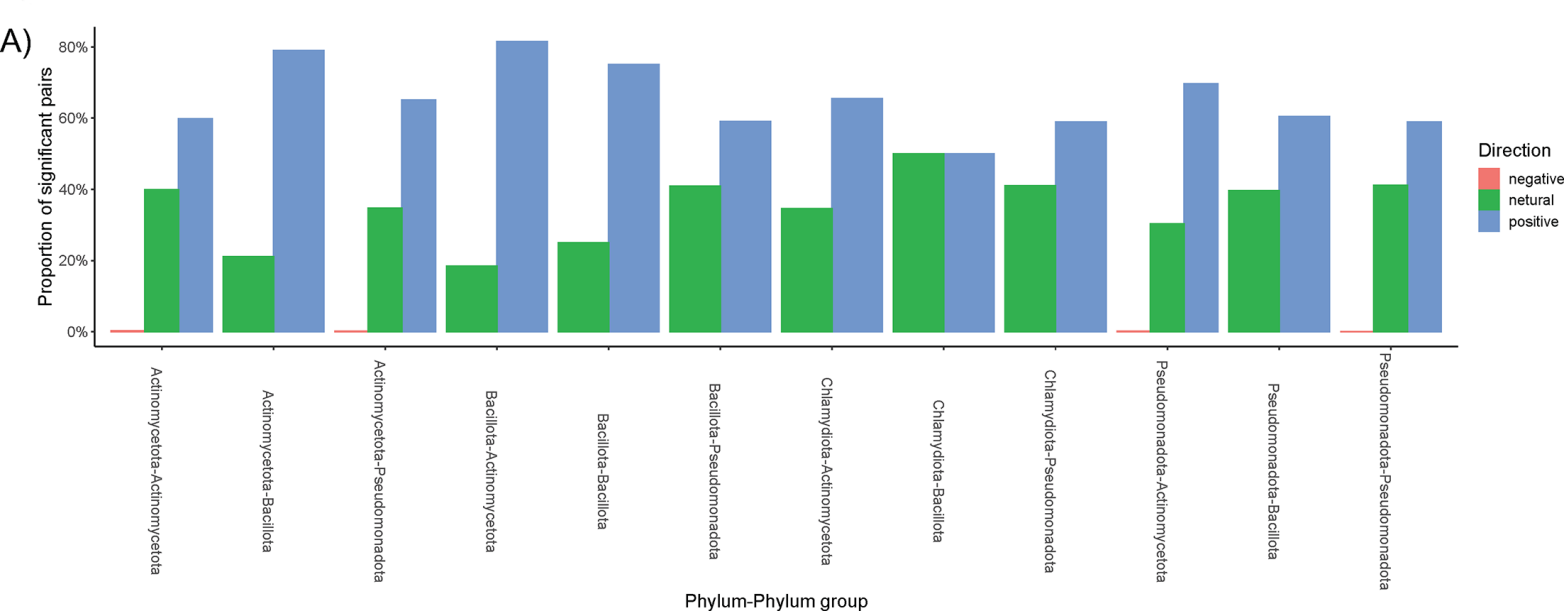
Selection pressure analysis of the frc gene. (A) Pairwise dn:ds ratios for the *frc* gene, based on high-throughput amplicon sequencing. All pairwise comparisons of *frc* ASVs were used to calculate dN/dS ratios. Shown are the distributions of negative, neutral, and positive dn:ds ratios, demarcated by all phylum:phylum pairs.

Examination of all pairwise dN/dS ratios reveals that, overall, the *frc* gene is heavily biased toward a positive, or radiative, selection, which is consistent with the molecular clock phylogenetic analysis (Fig. 7). The strongest selection pressures were between the Actinomycetota and Bacillota phyla, as well as within the Bacillota phylum. The least selection pressure is seen between the Chlamydiota and Bacilliota phyla.

### Sequence similarity networks

**Fig 8:**
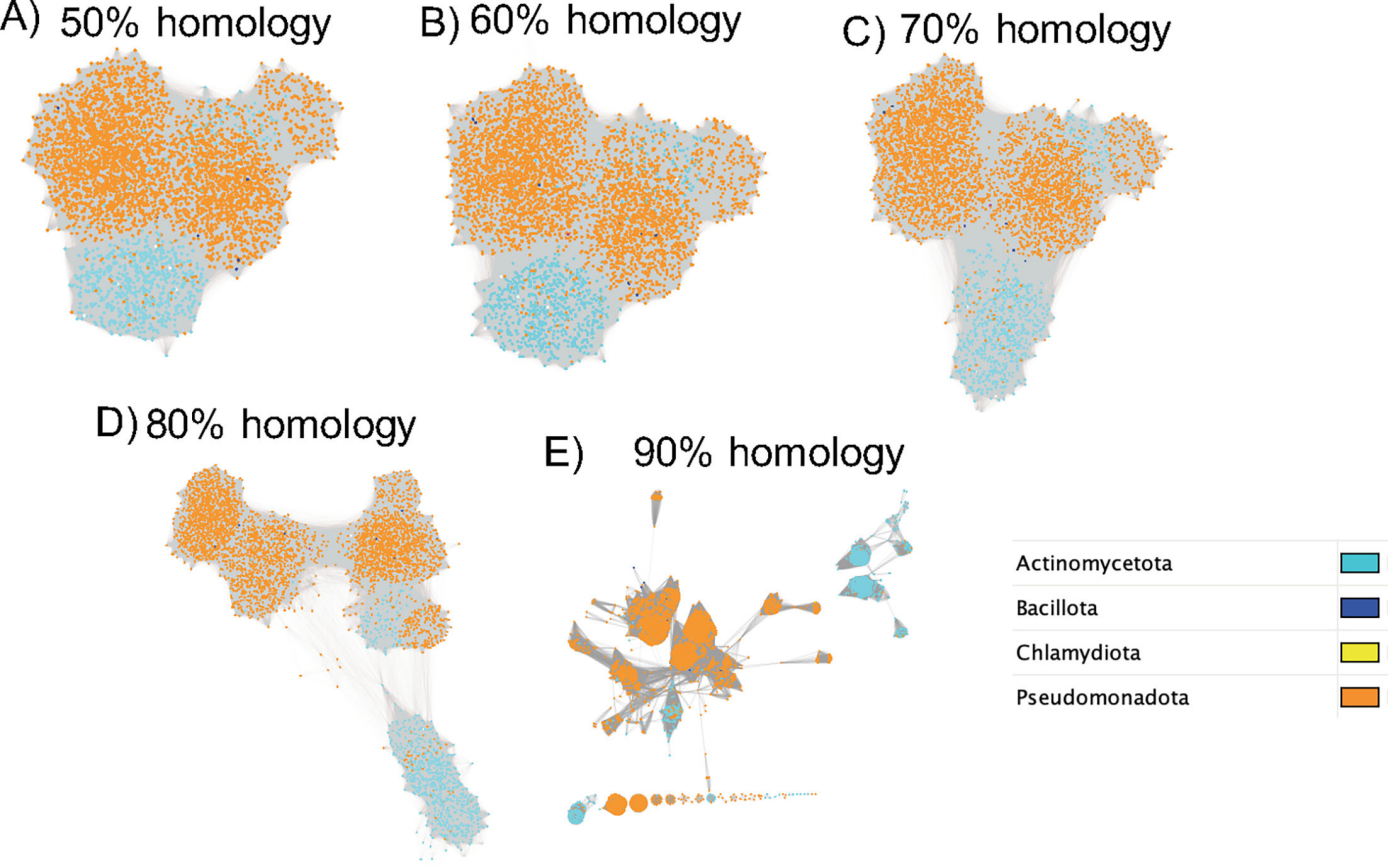
frc gene ASV sequence similarity networks. (A–E) SSN of *frc* ASVs from high-throughput amplicon sequencing at homology cutoffs ranging from 50% to 90%. Each dot represents an ASV, colored by phylum. Connections represent pairwise sequence homologies greater than the listed homology cutoff.

From the metagenomic derived *frc* gene ASVs, a single contiguous cluster was apparent through 80% sequence homology although clear sub-clusters formed and progressively separated with greater homology cutoffs. Sub-clusters were largely demarcated along phylum-level taxonomic lines (Fig. 8).

**Fig 9:**
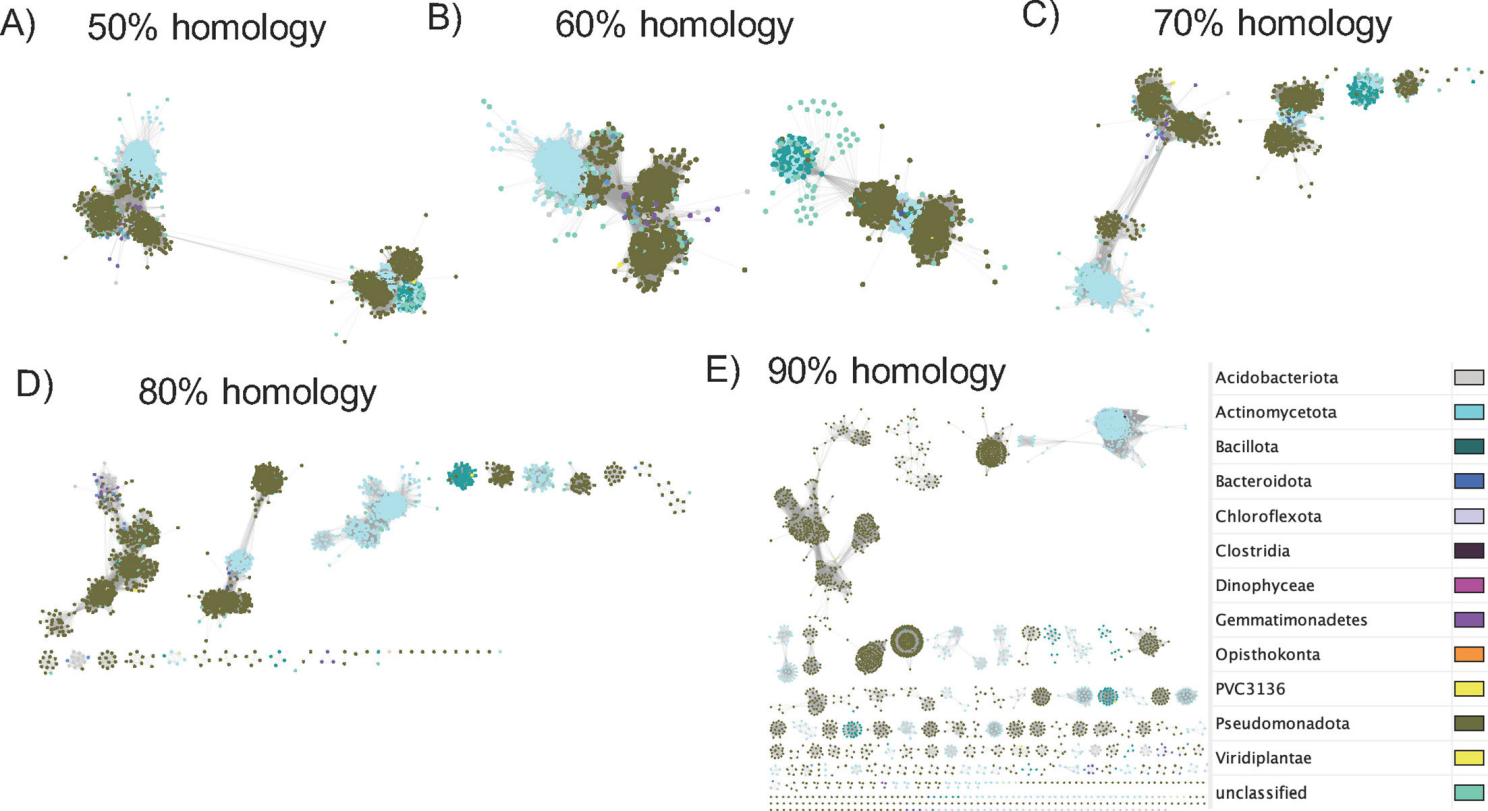
*frc* gene UniProt sequence similarity networks. (A–E) SSN of full length *frc* gene sequences from the UniProt database at homology cutoffs ranging from 50% to 90%. Each dot represents a gene sequence, colored by phylum. Connections represent pairwise sequence homologies greater than the listed homology cutoff.

In contrast to the SSN’s constructed from the metagenomic-derived *frc* gene sequences, those constructed from the full length, UniProt-derived *frc* gene sequences formed a single contiguous cluster at 50% homology, but split into two clusters at 60%, four clusters at 70%, and multiple clusters at 80% and 90% (Fig. 9). Again, clusters were largely demarcated by phylum-level taxonomy. However, when UniProt sequences were trimmed to the amplicon level, which is a more accurate comparison to our *frc* amplicon SSN networks, many homologous clusters were apparent, even at 50% homology (data not shown).

## DISCUSSION

The objectives of this study were to examine oxalate metabolism in the gut through an ecology and evolution perspective, to delineate hypotheses that evolutionary processes occurred at either the gene or taxon level. Results show that the level of redundancy in oxalate metabolic pathways within the gut is far greater than previously realized, with multiple pathways encoded by different genes distributed across diverse taxa (Fig. 2), consistent with past studies (57). Similar results were obtained for metabolic pathways that could utilize the by-products of oxalate metabolism, carbon dioxide, acetate, and formate (Fig. S1 to S4). The distribution of oxalate metabolism genes across 33%–45% species within the gut is indicative of a strong historical pressure to develop this function, consistent with a constant influx of oxalate through the diet. However, the proportion of the gut microbiota estimated to harbor genes for the metabolism of formate or carbon dioxide exceeds 95% across multiple pathways, indicative of a far greater selective pressure. In fact, formate and carbon dioxide can result from multiple host and microbial pathways (58, 59). Beyond the relevance to microbial ecology and evolution, oxalate and formate metabolism has considerable biomedical implications. For instance, oxalate is implicated in or a known causative factor for multiple chronic inflammatory conditions such as kidney stones, chronic kidney disease, vascular calcification, or cardiovascular disease calcium oxalate (60–70). In a previous study, which was the source of shotgun metagenomic data and genomes here, we found that oxalate and butyrate metabolism genes, as well as the *Faecalibacterium* genus, were significantly reduced in USD patients (28). In that study, the reduction of *Faecalibacterium* was thought to be primarily important for the production of butyrate. However, in the current analysis, we show that the *Faecalibacterium* genomes also harbored genes for oxalate and/or the AMSF pathways. Importantly, those genomes containing oxalate-degrading genes were twice as likely to be reduced in USD patients than those without (Fig. 3D, based on FDR < 0.05 from DESeq2 differential abundance analysis), which suggests that these bacteria may also help protect against USD through oxalate metabolism as well as butyrate synthesis.

In the current study, we probed hypotheses on gene vs taxon selection through multiple avenues and independent populations. Based on analysis of 151 full-length genomes derived from a cohort of 70 human participants, we found that bacteria from the Bacilliota phylum harbored the most genes per genome, on average (Fig. 2C). However, while some oxalate metabolism genes, such as succinyl-CoA transferase and oxalate-binding proteins, were broadly found in multiple phyla, others, such as formyl-CoA transferase and the oxalate-formate transporter, were primarily relegated to a single phylum (Pseudomonadota). It must be noted that while oxalate-handling genes were found in 32.5% of genomes, only 3.9% of genomes had the *frc* gene specifically. In genome-based analyses, 67% of genomes with the *frc* gene came from the Pseudomonadota, while the remaining originated from Bacilliota bacteria. Importantly, in an independent human population, the Pseudomonadota phylum comprised 1.75% of the gut microbiota, but 19.62% of the *frc* abundance and 82% of the unique *frc* variants, based on amplicon sequencing (Fig. 5; Fig. S5B). Similar patterns of gene distributions along phylum lines were also apparent in acetogenic, sulfate-reducing, and formate oxidation pathways (Fig. S1, S3, and S4). However, genes for methanogenesis exhibited a more consistent distribution between phyla (Fig. S2). Collectively, these data also support the taxon-level selection hypothesis.

In contrast to the above data, we found that genes encoding a single OAMSF function exhibited much greater co-occurrence than those that encoded multiple functions, indicative of potential metabolic synergy among single function genes (Fig. 4C). The exception to this were genes that could perform in both oxalate and acetogenic pathways, which exhibited the highest level of co-occurrence. Very little co-occurrence was identified when data were mapped to genomes, with the exception of genomes that contained genes for all or most of the OAMFS pathways. When looking at a single gene, *frc,* through sequence similarity network analysis, we found that the radiation of this gene was largely demarcated along phylum-level lines (Fig. 8 and 9), in support of taxon-level selection. However, a deeper analysis of that data suggests some nuance is apparent. Specifically, we see that the UniProt *frc* gene networks exhibited less homology than metagenomic derived genes (Fig. 8 and 9, based on sequence similarity network analysis). Data suggest that the within population divergence (genes derived from metagenomics of 70 participants) is lower than multiple population divergence (genes derived from multiple sources uploaded to UniProt), suggestive of horizontal gene transference. Thus, SSN data reflect a degree of gene-level selection as well. Finally, while the *frc* gene was largely relegated to the Pseudomonadota phylum, RelTime analysis (Fig. 6) at the genus level suggests multiple radiation events, whereby the ancestoral form of the gene first evolved in *Bradyrhizobium,* which then transferred to multiple other genera, indicative of gene-level selection. The overall positive selection pressure seen in thedN/dS ratios supports this hypothesis (Fig. 7). Importantly, the *Bradyrhizobium* comprised 0.004% of the gut microbiota in our human population, based on 16S rRNA sequencing, but 2.73% of the frc abundance and 26% of the unique *frc* variants, further supporting this hypothesis. *Bradyrhizobium* is a common soil bacterium with species that can use oxalate as a sole carbon and energy source (71). Subsequently, it appears that multiple radiation events have occurred over time, with some taxa, such as *Escherichia* or *Streptomyces,* acquiring the gene once, and other taxa, such as *Klebsiella,* or *Methylbacterium,* acquiring the gene multiple times, through currently unknown mechanisms. These data suggest variability along genus lines for the ability to receive genes through horizontal gene transfer events. When taken together, our data suggest that oxalate exposure promotes selection at the gene level, which is constrained by taxonomic divergence.

Beyond delineating hypotheses on gene- vs taxon-level evolutionary selection pressures, our data also offer insight into the emergence of ecological specialization or generalization. Generalist species are those who utilize a wide variety of resources to maximize their adaptability to environmental fluctuations. In contrast, specialist species are those who can effectively exploit a consistently available, but difficult to access, resource as a means of gaining a competitive advantage (8). For molecules that can differentially act as inhibitory toxins or a carbon and energy source, organisms can evolve to eliminate the toxin, such as oxalate or formate, from the environment as a means to remove its inhibitory impact on themselves, termed bioremediation (72). On a species level, while *Bradyrhizobium,* which appears to harbor the ancestral form of *frc,* can utilize oxalate as a sole carbon and energy source, it can also utilize other molecules as resources, indicative of a generalist (71). In contrast, *Oxalobacter formigenes,* which is a well-known oxalate-degrading specialist, appears to have acquired the *frc* gene once, relatively recently. These data suggest a sequence of events whereby a generalist soil bacterium acquired the ability to utilize abundantly available oxalate, which is commonly produced by plants to deter herbivory (10). These soil microbes were likely ingested by many plant-eating animals and became a part of the gut microflora. Plant-consuming animals would see high oxalate exposures, which, given the inhibitory effects of oxalate on bacterial growth, there would be a strong selection pressure for the bioremediator phenotype for oxalate. With the radiation of oxalate-degrading genes across taxa, combined with continued oxalate exposure, the stage is set for key species, such as *O. formigenes,* to repurpose these bioremediation genes to exploit oxalate for the purposes of growth. Such a specialist function would give this species a selective advantage, with little competition, given that the high amounts of oxalate degradation to support the growth of a specialist species would not only remove an environmental toxin, but would also lower the energetic requirements of bioremediation for other oxalate-degrading species. On a gene level, we found that nearly all of the genes (92%) examined were present in only one of the OAMFS pathways. This is compared to 2.6% of genomes that harbored genes for only one pathway (Fig. 4A). These data suggest that the species-level acquisition of genes to support ecological generalism is evolutionarily easier than adapting existing genes to multiple metabolic pathways.

In conclusion, results from our study show that oxalate and formate metabolism is highly redundant in the gut microbiota. While genes for oxalate metabolism span diverse taxa, specific genes/pathways are taxonomically restricted. Molecular clock analysis suggests radiation from generalist oxalate users, such as *Bradyrhizobium*, to bioremediating bacteria, and finally to oxalate specialists such as *O. formigenes*. Sequence similarity analyses of metagenomic and UniProt data sets suggest that *frc* gene radiation in the gut is driven more by horizontal gene transfer events rather than divergence within species-level populations. Collectively, data provide robust evidence supporting the hypothesis that oxalate exposure puts selective pressure at the gene level, but that the radiation of genes for oxalate metabolism is restricted by taxonomic divergence, particularly at the phylum level. Results provide fundamental insight into evolutionary processes within the gut microbiota and have biomedical implications when it comes to developing bacteriotherapies designed to promote or inhibit specific microbial functions.

## Data Availability

Data have been made publicly available with accession numbers SAMN38839607–SAMN38839718 and SAMN38777353–SAMN38777654.

## SUPPLEMENTAL MATERIAL

Supplemental material msphere.00913-24-s0001.docx Supplemental figures.

ASM does not own the copyrights to Supplemental Material that may be linked to, or accessed through, an article. The authors have granted ASM a non-exclusive, world-wide license to publish the Supplemental Material files. Please contact the corresponding author directly for reuse.
