# Supplementary material for: Metagenomic and phylogenetic analyses reveal gene-level selection constrained by bacterial phylogeny, surrounding oxalate metabolism in the gut microbiota
Source: mSphere. 2025 May 13;10(6):e00913-24. doi: 10.1128/msphere.00913-24 (PMC12188714; doi:10.1128/msphere.00913-24)
Supplement: Supplemental material — Supplemental figures. [file msphere.00913-24-s0001.docx]

**Supplemental Figure legends**

***Figure S1: Acetogenic gene distribution in a human population:*** A) Distribution of acetogenic gene number from 152 genomes based on metagenomic data from human stool samples. B) Acetogenic gene distribution in the 152 bacterial genomes, by phylum. C) Average number of acetogenic genes in each genome by genus.

***Figure S2: Methanogenic gene distribution in a human population:*** A) Distribution of methanogenic gene number from 152 genomes based on metagenomic data from human stool samples. B) Methanogenic gene distribution in the 152 bacterial genomes, by phylum. C) Average number of methanogenic genes in each genome by genus.

***Figure S3: Sulfate-reducing gene distribution in a human population:*** A) Distribution of sulfate-reducing gene number from 152 genomes based on metagenomic data from human stool samples. B) Sulfate-reducing gene distribution in the 152 bacterial genomes, by phylum. C) Average number of sulfate-reducing genes in each genome by genus.

***Figure S4: Formate gene distribution in a human population:*** A) Distribution of formate gene number from 152 genomes based on metagenomic data from human stool samples. B) Formate gene distribution in the 152 bacterial genomes. C) Average number of formate genes in each genome by genus.

***Figure S5: Taxonomic profiles of the 16S rRNA and* frc *genes in data derived from mouse studies:*** A,B) Phylum (A) and Genus (B) profiles of the 16S rRNA gene.
